# Simulation of Friction Stir Welding of AZ31 Mg Alloys

**DOI:** 10.3390/ma17204974

**Published:** 2024-10-11

**Authors:** Sili Feng, Zhe Liu, Renlong Xin

**Affiliations:** 1 College of Materials Science and Engineering, Chongqing University, Chongqing 400044, China; fsl1503040209@163.com; 2 Guangdong Provincial Key Laboratory of Material Joining and Advanced Manufacturing, China-Ukraine Institute of Welding, Guangdong Academy of Sciences, Guangzhou 510650, China; 3 Shenyang National Laboratory for Materials Science, Chongqing University, Chongqing 400044, China

**Keywords:** friction stir welding, moving heat source model, weld temperature, Mg alloy

## Abstract

Friction stir welding has been extensively applied for the high-quality bonding of Mg alloys. The welding temperature caused by friction and plastic deformation is essential for determining the joint characteristics, especially the residual stress and weld microstructure. In this work, a modified moving heat source model was proposed by considering the variations in heat generation caused by friction shear stress at both the side and bottom surfaces of the tool. The application of this model was further extended to the entire welding process, especially in the plunging stage. The relative errors between the experimental and simulated peak temperatures at characteristic points were small, with a maximum of 10%, thereby validating the model for accurate temperature prediction. Furthermore, the influence of welding and rotational speed on temperature fields was systematically investigated. At relatively low welding and rotational speeds, the welding temperature increased significantly with either an increase in rotational speed or a decrease in welding speed. However, this effect gradually diminished at higher welding and rotational speeds. These results provide some valuable guidelines for controlling heat generation to improve the quality of Mg alloy welds.

## 1. Introduction

Mg alloy is considered the lightest metallic structural material, and has received increasing attention in the aerospace and automobile industries due to its exceptional strength-to-weight ratio [1,2,3,4]. Welding is an essential manufacturing technique employed for the production of engineering components. As a solid-state joining technique, friction stir welding (FSW) can avoid surface distortion and grain coarsening, which is particularly suitable for the welding of light metals such as Mg alloy [5,6,7]. The heat generated during FSW process is mainly attributed to the friction between the welding tool and workpiece, and the plastic deformation occurs around the tool. It has been recognized that the amount of heat generation is predominantly affected by welding parameters, such as welding speed, rotational speed and the axial compressive force [8,9,10]. Controlling the heat generation during the welding process can affect the distribution of residual stress, the microstructure evolution and the mechanical properties of the joints [11,12,13]. Therefore, it is essential to perform a comprehensive assessment of the temperature field generated in the workpiece during FSW.

Considerable efforts have been devoted to understanding the workpiece temperature during FSW through either experimental measurement or numerical simulation. The thermal cycle at some pre-selected points of a workpiece can be obtained during the FSW process by temperature measuring equipment. However, due to the limitations of measurement equipment, it is not possible to obtain the distribution of temperature across the entire workpiece. By contrast, several finite element approaches, including the arbitrary Lagrangian–Eulerian model (ALE) [14,15], the coupled Eulerian–Lagrangian model (CEL) [16,17,18] and the smoothed particle hydrodynamics model (SPH) [19,20,21], have been developed to simulate the whole temperature field of a workpiece during FSW. It is agreed that the complex coupling effect between fluid dynamics and solid mechanics considered in these thermo-mechanical or thermo-flow coupled models requires expensive computational resources. Moreover, the immediate effect of welding parameters on heat generation during FSW cannot be or has not been examined by these models, which hinders the improvement in welding quality by controlling the heat generation during the welding process.

To address the aforementioned limitations, an appropriate heat source moving model should be proposed to accurately simulate the FSW process [22,23,24] with less time. With a precise description of both the heat generation and heat flux, the model can effectively simulate the characteristics of the temperature field across the workpiece during FSW. In previous studies, three contact states at the tool/workpiece interface were considered to estimate the heat generation, namely sliding, sticking and partial sliding/sticking [25]. When the velocity of the base material is zero, the contact condition is regarded as sliding, otherwise it is considered as sticking or partial sliding/sticking. In previous studies, a certain axial compressive force and effective friction coefficient were often selected with the assumption of the sliding contact condition. However, it is known that the axial compressive force is dependent on the welding parameters and welding stage. Pankaj et al. [26] found that the axial compressive force exhibited an initial rise, followed by a subsequent drop, and remained relatively steady during the stable welding stage. Kamruan et al. [27] further revealed that the axial compressive force could increase by increasing the rotation speed or decreasing the shoulder diameter. Nevertheless, it is agreed that accurately estimating the axial compressive force is challenging and the aforementioned heat generation model often leads to an overestimated temperature field for the FSW process. Alternatively, under the assumption of a sticking condition, heat generation can be assessed through the friction shear stress calculated by multiplying the tensile yield stress by a factor of $\sqrt{3}$. Liu et al. [28] applied this theory to develop a moving heat source model for understanding the temperature field during FSW. However, considering the heat generation of the shoulder and the pin as a simple distribution of the total heat cannot accurately reflect the complex heat distribution in reality. Recently, a hybrid heat generation model, combining the aforementioned two models, has been employed for welding tools with different surfaces. Based on the velocity of the contact point relative to the tool at the workpiece surface [25], the contact state at the bottom of the tool and workpiece interface was considered as a sliding condition, whereas the contact along the side of the tool and workpiece interface was regarded as either a sticking condition or a partial sliding/sticking condition. Alhourani et al. [29] calculated the heat generation at the bottom and side surface of the stirring pin for the stable welding stage based on the axial compressive force and the friction shear stress. Song et al. [30] further employed the axial compressive force to determine heat generation at the shoulder bottom. Previous studies have indicated that it is more reliable to determine heat generation by considering both the axial compressive force and friction shear stress. However, previous studies have primarily focused on the temperature field simulation of Al alloys during the stable welding stage of FSW, with less attention given to the simulation of Mg alloys and the heat generation variations during the plunging stage. The material properties and the plunging stage both influence the overall temperature field during FSW. Therefore, more sophisticated simulation work is needed that considers the effects of different welding stages and heat-generating parts on the overall heat generation.

In this work, a modified moving heat source model is employed for calculating the heat generated in an AZ31 Mg alloy during FSW. The accuracy of this new model in simulating the temperature caused by FSW and the dependence of simulated temperature gradients on welding parameters in different welding stages were validated via experimental measurements. The findings in this work might provide some insights into optimizing welding parameters and controlling heat generation to improve the quality of Mg alloy welds.

## 2. Experimental Procedures

**Figure 1:**
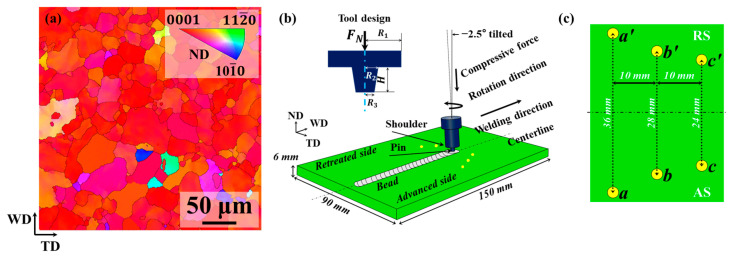
(**a**) Orientation map of commercial AZ31 Mg alloys. Schematic illustrations of (**b**) FSW process and (**c**) temperature measurement.

In this study, the selected model material was a 6 mm thick commercial AZ31 Mg alloy sheet with an approximate size of 150 mm × 90 mm. The microstructure of the base material (BM) was characterized by electron backscattered diffraction (EBSD) using a TESCAN system. The corresponding orientation map is illustrated in Figure 1a. The estimated mean grain size was found to be approximately 18.5 μm. After being polished with abrasive paper and cleaned with ACII acetone, FSW was employed on the AZ31 plates using an RSW-1610-6T-2D CNC gantry friction stir welding machine from RuiSong Technology, Guangzhou, Guangdong Province, China. Figure 1b demonstrates the schematic of the welding procedure. The primary directions of FSW geometry are defined as normal direction (ND), transverse direction (TD) and welding direction (WD). The equipped non-consumable tool with a shoulder diameter of 18 mm and a conical threaded pin with a toe diameter of 8 mm, a tip diameter of 5.5 mm and a length of 5.6 mm was used for the FSW process. The tool part was angled 2.5° away from the WD and rotated in a counter-clockwise direction at a constant rotational speed of 800 rpm. The entire welding process consists of four stages, depending on the feeding speed and dwelling time: (1) the plunging stage with a slow plunging speed of 20 mm/min; (2) the dwelling-I stage with a dwelling time of 6 s; (3) the stable welding stage at a welding speed of 90 mm/min; and (4) the dwelling-II stage with a dwelling time of 2 s. In addition, temperature measurements were conducted using K-type thermocouples (see Figure 1c). To more accurately capture the thermal cycle curves at the characteristic points during the welding process, the sampling frequency for temperature measurement was 10 Hz, and the temperature data were recorded to three decimal places. During the subsequent validation, only the first significant digit of the temperature data will be used. The measurement positions were labelled as a, b, c, a’, b’, c’ with each hole having a diameter of 1 mm and a depth of 2 mm. In particular, positions a, b and c were placed on the advanced side (AS), whereas positions a’, b’ and c’ were located on the retreated side (RS). The distances from the weld centre to a and a’ were 18 mm, to b and b’ were 14 mm, and to c and c’ were 12 mm. The distance between each adjacent point was 10 mm. Additionally, the initial temperatures measured by K-type thermocouples at points a, a’, b, b’, c and c’ were approximately 36.252 °C, 36.427 °C, 36.328 °C, 36.231 °C, 36.259 °C and 36.280 °C, respectively.

**Figure 2:**
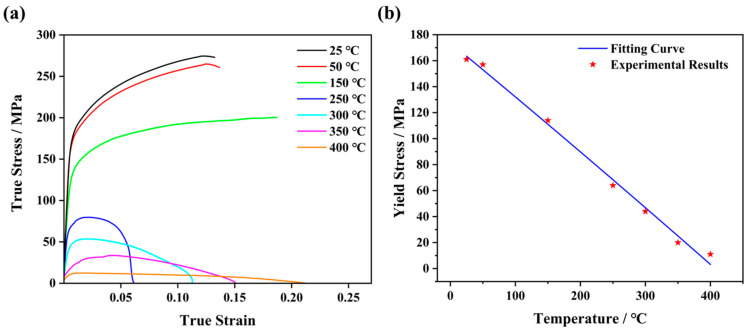
(**a**) Tensile stress–strain curves of AZ31 Mg alloys at elevated temperatures. (**b**) Variations in yield strength as a function of tensile temperature at a strain rate of 10^−3^ s^−1^.

To precisely describe the thermal deformation response, tensile deformation experiments were performed at various temperatures. Dog-bone-shaped tensile samples, with a nominal gage size of 110 mm × 20 mm × 2 mm, were extracted from the BM. With a strain rate of 1 × 10^−3^ s^−1^ along the TD, three replicates of the transverse tensile test were performed to guarantee representative results, as shown in Figure 2. It is demonstrated that the elevated temperature leads to a reduction in both ultimate tensile strength and yield strength. Furthermore, the relationship between yield stress and temperature was determined using the least-square approach to evaluate the regression equation and its corresponding regression coefficients (see Figure 2b):

(1) $$\sigma_{s}=1.45\times 10^{9}-33\times {\left(6233.07+T\right)}^{2}$$

where $\sigma_{s}$ (Pa) represents the yield stress and T (°C) denotes the temperature.

## 3. Moving Heat Source Model for FSW

**Figure 3:**
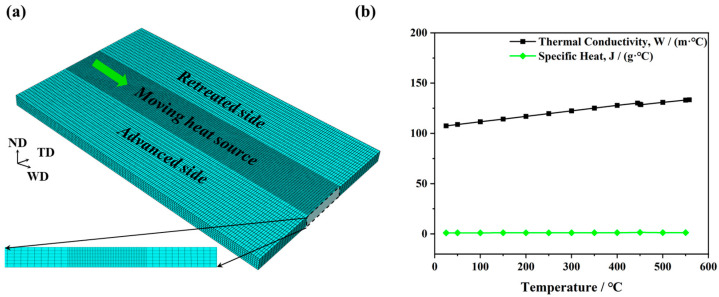
(**a**) Model configuration of FSW simulation. (**b**) Thermophysical parameters of AZ31 Mg alloys.

In this study, the analytical model was applied with the commercial finite element analysis software ABAQUS 6.14 to estimate heat generation and the associated temperature field during FSW. The detailed mesh generation of a 6 mm thick plate is presented in Figure 3a. In order to strike a balance between computational efficiency and accuracy, two types of mesh size of 0.5 mm × 1.5 mm × 2 mm and 1 mm × 1.5 mm × 2 mm (ND × WD × TD) were employed in the weld zone and the AS/RS, respectively. In total, the workpiece consists of 72,900 three-dimensional 8-node linear elements (DC3D8). In a previous work [31], two specific welding parameters were used: a rotation speed of 800 rpm with a welding speed of 90 mm/min, and a rotation speed of 1600 rpm with a welding speed of 600 mm/min. In order to determine the influence of welding parameters on temperature distribution, a thermal simulation of the FSW process was performed at a welding speed and rotational speed ranging from 90 mm/min to 600 mm/min and from 800 rpm to 1600 rpm, respectively. Details of the welding parameter combinations used in the model are listed in Table 1.

### 3.1. Governing Equations and Boundary Conditions

The governing equation of thermal simulation in the FSW process is given as follows:

(2) $$\rho c\frac{\partial T}{\partial t}-\nabla \left(k\nabla T\right)=q$$

where ρ represents the material density (1780 kg/m^3^ in this research), c denotes the material specific heat, k indicates the material thermal conductivity, and q represents the heat flux [32,33]. It is established that both specific heat and thermal conductivity are sensitive to temperature. Figure 3b illustrates the relationship between the thermophysical parameters of AZ31 Mg alloy and temperature. It is revealed that the value of thermal conductivity climbs from 107.5 W/(m·°C) at 25·°C to 133.4 W/(m·°C) at 560·°C. In contrast, the specific heat keeps relatively stable. When the temperature is 25·°C, the value of specific heat is 1.02 J/(g·°C), and when the temperature is 550·°C, the value of specific heat is 1.27 J/(g·°C).

Moreover, the workpiece surfaces are subjected to the following convective boundary conditions. The thermal convection coefficient between the Mg bottom surface and the steel backing plate was 300 W/(m^2^·°C). By contrast, the natural convection between residual surfaces of the workpiece and the surrounding air was characterized by a low heat convection coefficient of 30 W/(m^2^·°C). Additionally, the environment temperature was set to 35·°C. Given the brief duration of the FSW process, the impact of heat radiation on the temperature field was neglected in the thermal simulation.

### 3.2. Heat Generation at Different Stages of FSW Process

**Figure 4:**
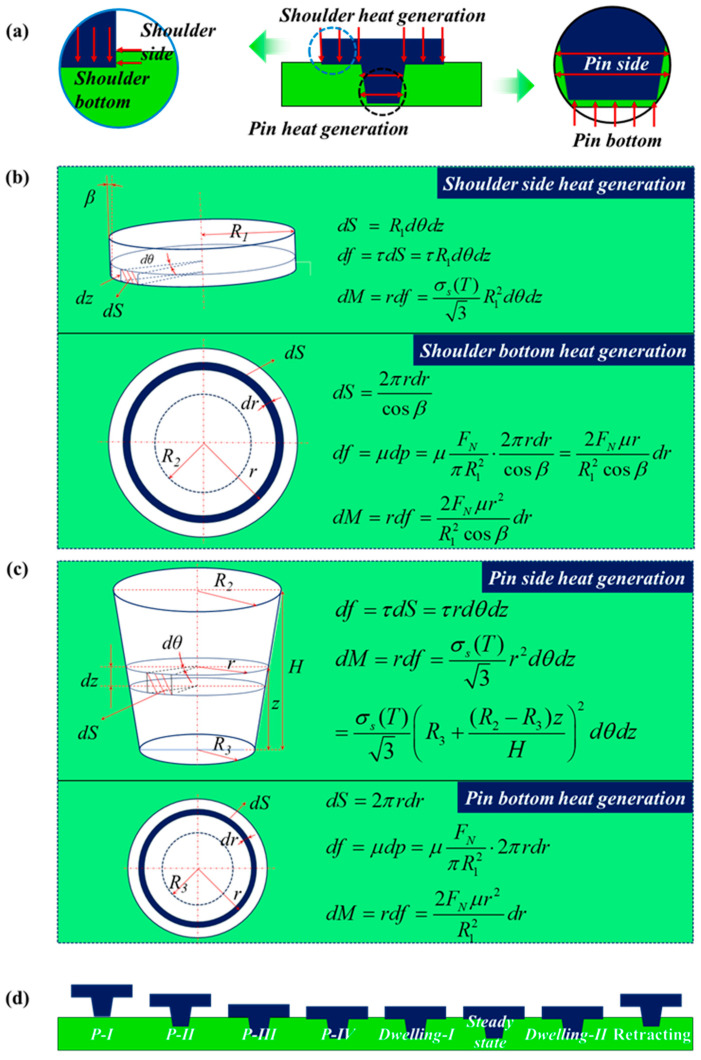
Schematic of (**a**) heat generation originating from the tool shoulder (see (**b**)) and pin (see (**c**)), (**d**) substages of the entire FSW process.

Figure 4a–c illustrate that the friction at the tool and workpiece interface is the primary source of heat generation (Q). The contact interface can be further divided into a shoulder/workpiece interface and pin/workpiece interface. Specifically, the heat generation at the shoulder ($Q_{s}$) includes two components, i.e., that from the shoulder bottom ($Q_{sb}$) and from the shoulder side ($Q_{ss}$). Similarly, the heat generation from the pin ($Q_{p}$) is equivalent to the sum of that caused at both the pin bottom ($Q_{pb}$) and the pin side ($Q_{ps}$). Consequently, the total heat generation from the stirring tool can be calculated as:

(3) $$Q=Q_{s}+Q_{p}=Q_{sb}+Q_{ss}+Q_{pb}+Q_{ps}$$

To estimate the respective quantity of heat, a general formula is given as an infinitesimal expression [28]:

(4) dQ=ωdM=ωrdf

where ω represents the tool angular velocity, r indicates the distance from the heat source centre, and dM and df denote the torque and the frictional shear force for an infinitesimal segment area, respectively. By assuming a sliding interface between the tool bottom and workpiece, the corresponding shear stress can be calculated as follows:

(5) df=μdp

where μ represents the friction coefficient, and dp denotes the contact pressure of an infinitesimal segment area. By contrast, the estimated shear stress between the tool side and the workpiece corresponds to the yield shear stress $\tau_{yield}$ with the contact assumption of the sticking condition. Thus, the corresponding shear stress can be calculated as follows:

(6) $$df=\tau_{yield}dS=\frac{\sigma_{yield}}{\sqrt{3}}dS$$

where $\sigma_{yield}$ represents the yield stress of workpiece materials, and dS is the area of an infinitesimal segment.

During the whole welding process, the interaction between the stirring tool and the workpiece varies depending on the plunge depth. The corresponding formula for heat generation would inevitably change. As illustrated in Figure 4d, the welding procedure is segmented into five periods: plunging stage, dwelling-I stage, stable welding stage, dwelling-II stage and retracting stage. During the final retracting stage, the disengagement between the stirring tool and the workpiece causes an interruption in heat generation. The residual stages can be further categorized into two types: (1) Type I, the stirring tool is in full contact with the workpiece, which corresponds to the dwelling-I stage, stable welding stage and dwelling-II stage; (2) Type II, the stirring tool exhibits a partial contact with the workpiece, i.e., the plunging stage.

(1) Type I heat generation

The computation of type I heat generation can be described as:

(7) $$Q_{sb}=\omega M_{sb}=\omega \int_{R_{1}}^{R_{2}}\frac{2F_{N}\mu r^{2}}{{R_{1}}^{2}\cos\beta }dr=\frac{2\omega F_{N}\mu (R_{1}^{3}-R_{2}^{3})}{3R_{1}^{2}\cos\beta }$$

(8) $$Q_{ss}=\omega M_{ss}=\omega \int_{0}^{2\pi }d\theta \int_{0}^{h}\frac{\sigma_{s}(T)}{\sqrt{3}}R_{1}^{2}dz=\frac{2\sqrt{3}\omega \pi hR_{1}^{2}\sigma_{s}(T)}{3}$$

(9) $$Q_{pb}=\omega M_{pb}=\omega \int_{0}^{R3}\frac{2F_{N}\mu r^{2}}{R_{1}^{2}}dr=\frac{2\omega F_{N}\mu R_{3}^{3}}{3R_{1}^{2}}$$

(10) $$Q_{ps}=\omega M_{ps}=\omega \int_{0}^{2\pi }d\theta \int_{0}^{H}\frac{\sigma_{s}(T)}{\sqrt{3}}{\left(R_{3}+\frac{(R_{2}-R_{3})z}{H}\right)}^{2}dz=\frac{2\sqrt{3}}{9}\pi \omega \sigma_{s}(T)H(R_{2}^{2}+R_{2}R_{3}+R_{3}^{2})$$

where $R_{1}$ represents the shoulder radius, while $R_{2}$ and $R_{3}$ denote the tip radius and toe radius of the pin, respectively. $F_{N}$, β, h and H are the axial compressive pressure of the tool, the tilt angle of the shoulder bottom, the plunge depth of the shoulder and the height of the pin, respectively.

As mentioned before, the axial compressive force exerted by the stirring tool is influenced by rotational speed [34,35]. Within the rotational speed range of 800–1600 rpm employed in this study, the axial compressive forces were assumed to be 5000, 4250 and 3500 N during the plunging stage, and 10,000, 8500 and 7500 N during the stable welding stage. It is reported [36] that AS is consistent with the tool’s rotation direction, and thus, more frictional heat is generated, while relatively less frictional heat is produced at RS. For Mg alloys, the friction coefficient typically ranges between 0.1 and 0.4 [37]. The applied force on the interface and the contact condition will influence the friction coefficient. In the present work, the friction action between the workpiece and tool were described by the adhesion friction mechanism [38], in which the friction coefficient is affected by junction areas between the two parts. The adhesion friction mechanism is typically defined as:

(11) $$\mu =\frac{1}{3{\left(k^{-2}-1\right)}^{\frac{1}{2}}}$$

where k is the ratio between the critical shear stress of the material at the contact interface and the critical shear stress of the material itself. It was assumed that k was 0.6 at AS and 0.5 at RS, respectively. Therefore, the corresponding value of μ was set as 0.25 at AS and 0.192 at RS, respectively.

(2) Type II heat generation

In terms of Type II heat generation, the plunging stage is subdivided into four substages, i.e., P-I, P-II, P-III and P-IV, which correspond to the sequential contact between the pin bottom and workpiece, the pin side and workpiece, the shoulder bottom and workpiece, and the shoulder side and workpiece, respectively. In particular, the penetration depths of both the shoulder and pin into the workpiece are evaluated by the plunging speed and plunging time. Therefore, the heat generation at the side surfaces of the tool is described by:

(12) $$\begin{array}{c} Q_{ss}=\frac{2\sqrt{3}\omega \pi h(t)R_{1}^{2}\sigma_{s}(T)}{3}\\ h(t)=v\cdot t,0\leq t\leq \frac{h}{v} \end{array}$$

(13) $$\begin{array}{c} Q_{ps}=\frac{2\sqrt{3}}{9}\pi \omega \sigma_{s}(T)H(t)(R_{2}^{2}+R_{2}R_{3}+R_{3}^{2})\\ H(t)=v\cdot t,0\leq t\leq \frac{H}{v} \end{array}$$

where h(t) and H(t) represent the penetration depth of both the shoulder and pin into the workpiece, respectively. v is the tool plunging speed. The force-bearing area of the pin is simplified as a circle with an average radius $R_{4}$, which is an average of $R_{2}$ and $R_{3}$, i.e., $R_{4}=\frac{R_{2}+R_{3}}{2}$. Hence, the heat generation at the bottom surfaces of the pin is defined by:

(14) $$Q_{pb}=\frac{2\omega F_{N}\mu R_{3}^{3}}{3R_{4}^{2}}$$

### 3.3. Heat Flux during FSW Process

Based on the above calculations, the heat flux can be determined by dividing the heat generation by the effective volume or area. Due to a relatively shallow penetration of the shoulder into the workpiece, heat flux can be equivalently treated as a surface heat source. By contrast, the heat flux from the pin can be equivalently regarded as a volumetric heat source due to its deeper penetration into the workpiece.

Throughout the welding process, the heat flux experiences dynamic fluctuations. Particularly in the plunging stage, the magnitude of the heat flux shifts in response to the plunging action along the opposite direction of ND. However, during the steady welding stage, the magnitude of heat flux remains constant with the change in its position. Therefore, the magnitude and spatial location of the heat flux can be described by:

(15) $$q_{s}=\frac{3Q_{s}R}{2\pi (R_{1}^{3}-R_{2}^{3})},R_{2}\leq R\leq R_{1}$$

(16) $$\begin{array}{c} q_{p}=\frac{Q_{p}}{\pi R_{4}^{2}H(t)},R\leq R_{i}\\ R_{i}=\frac{1}{2}\left(R_{3}+\frac{\left(R_{2}-R_{3})H(t\right)}{H}\right) \end{array}$$

(17) $$\begin{array}{c} R=\sqrt{{\left(x-x_{1}\right)}^{2}+{\left(y-y_{1}\right)}^{2}}\\ x_{1}=x_{0}+dx\\ y_{1}=y_{0}\\ dx=v_{w}\cdot t \end{array}$$

where $q_{s}$ and $q_{p}$ represent the heat flux caused by the shoulder and pin, respectively. R denotes a circle constraining the heat source. $x_{0}$($y_{0}$) and $x_{1}$($y_{1}$) are the tool coordinates at both the initial and welding positions along the WD. $v_{w}$ is the welding speed.

## 4. Results and Discussion

### 4.1. Model Verification

**Figure 5:**
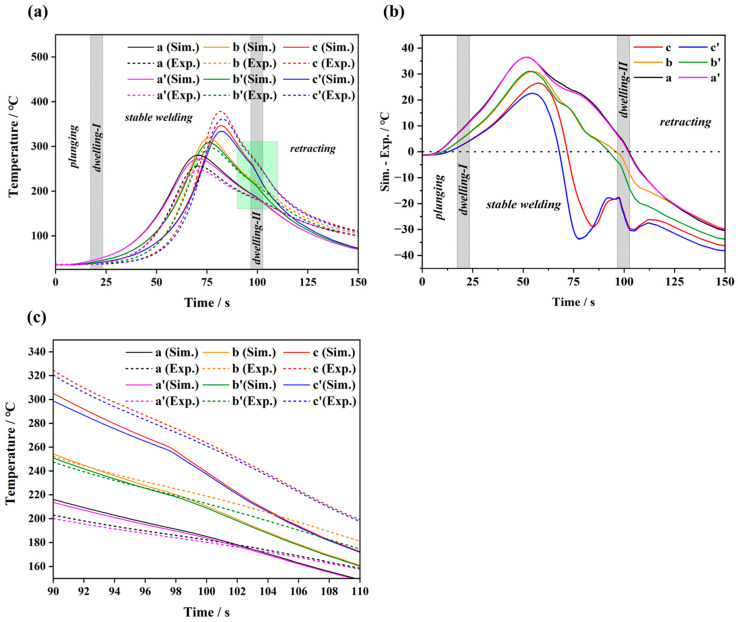
(**a**) Thermal cycle curves of characteristic measuring points originating from both simulation and experiment. (**b**) Simulation–experiment temperature difference. (**c**) Enlargement for local light-green region in (**a**).

The proposed thermal model was validated by recording the transient temperatures throughout the FSW process. Figure 5a demonstrates the thermal cycle curves of the characteristic measurement points from both simulations and experiments. The characteristic points selected in the simulation were the same as those in the experiment; specifically, the distances from the weld centre to a and a’ were 18 mm, to b and b’ were 14 mm, and to c and c’ were 12 mm. The distance between each adjacent point was 10 mm. Similar to the previous reports [17,18,39], all the curves show a similar pattern: an initial temperature increase is noted as the weld tool approaches the thermocouple, followed by a gradual decline as the distance between the measurement point and the tool increases. In particular, due to the distance difference from the measurement positions to the tool, the peak temperature on the AS gradually decreases from 378.4 °C at position c to 305.9 °C at position b and further to 256.5 °C at position a. A similar trend is also observed on the RS albeit with relatively lower values of peak temperature, i.e., 361.1 °C at position c’, 296.1 °C at position b’ and 247.2 °C at position a’. Therefore, the temperature differences at the same distance between the AS and RS are determined to be 17.3 °C, 9.8 °C and 9.3 °C, which is influenced by the generation and dissipation of heat. Furthermore, the peak temperatures from both the simulated and experimental results demonstrate a good consistency. The relative errors estimated from the experimental and simulated data of measurement points a, b and c are −8.4%, +4.3% and +9.4%, respectively. Additionally, the difference between the simulations and experiments is shown in Figure 5b. In the stable welding stage, a certain deviation is observed between the simulation and experimental results, with the simulation temperature being either higher or lower than the experimental temperature. This indicates that the model may have underestimated the heat dissipation or overestimated the heat input during this stage. However, the largest difference is below 40 °C, which is much smaller than the peak temperature. Overall, the simulation results are generally able to accurately replicate the temperature variation trends observed in the experiments. Thus, the validation of the moving heat source model established in this work is confirmed by a relatively small error of less than 10% at the corresponding points, as well as by the temperature trend shown in Figure 5. The relative error in our simulation is larger than that in previous studies which used the ALE or CEL methods to determine the temperature distribution of the workpiece during FSW. This larger error is primarily due to the fact that our model does not account for the effect of plastic deformation on the temperature. For instance, in previous studies [29], the temperature relative error between the simulation and experiment at characteristic points was less than 3%. However, most existing studies on temperature field simulations during FSW have focused on Al alloys, with comparatively few analyses involving Mg alloys. An interesting observation shows that the simulated temperature at position c is lower than the experimental temperature, while the simulated temperatures at positions a and b exceed the experimental values. This discrepancy arises from the differences in both the convective heat transfer coefficients and thermal conductivity of the workpiece between the simulation and real-world conditions. As illustrated in Figure 5c (the green shading in Figure 5a), the thermal cycle curves demonstrate a slight turning during the dwelling-II stage. This turning phenomenon is more noticeable at the measurement points in close proximity to the weld nugget, which originates from the zero-thermal generation during the retracting stage.

### 4.2. Temperature Field Distribution

**Figure 6:**
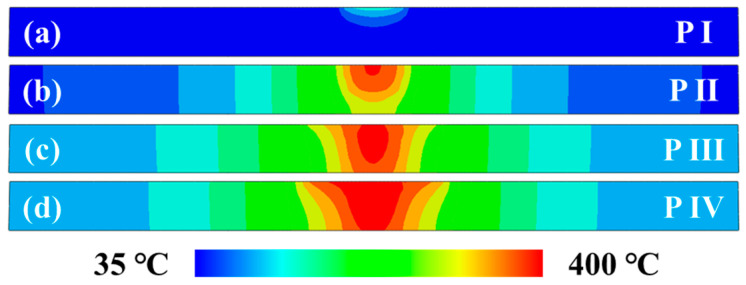
Simulated temperature fields in the transverse cross-section at various substages of the plunging stage: (**a**) P-I; (**b**) P-II; (**c**) P-III; (**d**) P-IV.

At the initial welding position, the estimated temperature profile in the cross-sectional region during the plunging stage is presented in Figure 6. With the plunging motion of a stirring pin, the peak temperature of the workpiece gradually increases and the corresponding high-temperature zone expands progressively. The same result was also observed by the most recent study carried out by Salloomi [16]. At the final step of the plunging stage, a simulated peak temperature of 394.2 °C is received on the workpiece surface. Due to the dynamic changes in heat generation, the resultant temperature fields exhibit significant alterations during the entire plunging stage. In the initial P-I step, the upper workpiece demonstrates a minor arc-shaped isothermal region, with a peak temperature of 149.9 °C. When the pin side surface starts to make contact with the workpiece, the isothermal area with an obviously higher peak temperature is detected at the P-II step. The shape is caused by a constrained region of heat flux. At the P-III and P-IV steps, a wider conical-shaped isothermal profile appears at the upper part of the workpiece due to the interaction between the workpiece and the stir pin shoulder.

**Figure 7:**
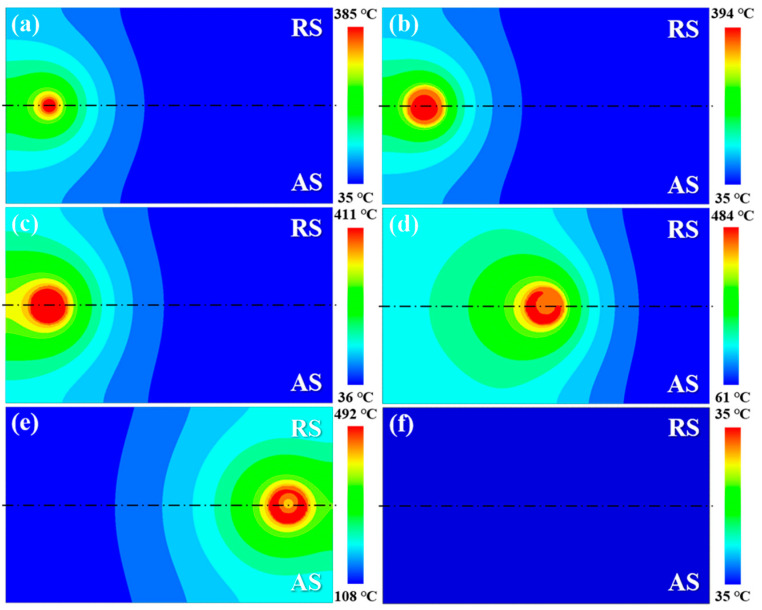
Simulated temperature fields in the upper surface at various substages of the FSW process: (**a**,**b**) plunging stage; (**c**) dwelling-I stage; (**d**) stable welding stage; (**e**) dwelling-II stage; (**f**) retracting stage.

Figure 7 depicts the temperature profiles on the upper surface of the workpiece across different welding stages. The high-temperature region displays a noticeable change with the movement of the heat source. At the initial weld position, the peak temperature reached 394.2 °C and 411.4 °C at the plunging and dwelling-I stage, respectively. The increase in peak temperature during the dwelling-I stage is attributed to the accumulation of heat generation at the same position. Given the constant heat generation caused by the friction at the tool/workpiece interface, the peak temperature remains at 484.5 °C throughout the stable welding stage and then increases to 492.2 °C at the dwelling-II stage. Furthermore, the temperature profiles on the AS and RS are asymmetric with respect to the weld centre due to the different heat generation at the two sides.

### 4.3. Effects of Welding Parameters on Temperature

**Figure 8:**
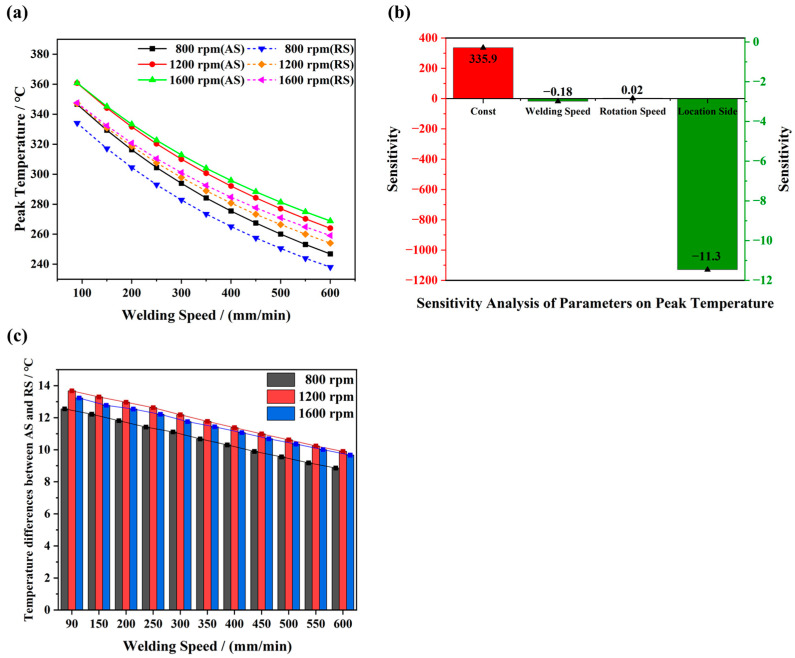
(**a**) Peak temperature simulated at points c (AS) and c′ (RS) with various welding parameters. (**b**) Sensitivity analysis of parameters on peak temperatures. (**c**) Peak temperature variations at the AS and RS.

It is widely recognized that the increased rotational speed combined with the reduced welding speed leads to a higher temperature. However, the intricate correlations between welding parameters and temperature fields are seldom addressed. Based on the discussion in the section ‘*Heat Generation at Different Stages of the FSW Process*’, it is clear that welding parameters directly influence heat generation. For instance, the relationship between rotation speed and heat generation is linear when other welding parameters are kept constant. However, although welding speed is not explicitly mentioned in the heat generation formulas, it also affects the temperature distribution. This is because a higher welding speed reduces the time that heat is applied to a particular area, which can decrease the temperature of the workpiece, whereas a lower welding speed allows more heat energy to accumulate, increasing the temperature. To investigate the relationship between the welding variables and temperature fields generated during the FSW process, the peak temperatures at positions c and c’ under various welding conditions are depicted in Figure 8. As shown in Figure 8a, the peak temperatures at both positions c and c’ exhibit a similar trend, i.e., a decrease in welding speed leads to a higher peak temperature at a constant rotational speed. Due to the less efficient heat dissipation at the higher welding speeds, the decrease in peak temperature becomes less pronounced as the welding speed increases. Furthermore, the peak temperature rises with increasing rotational speed in the low rotational speed range (800 rpm to 1200 rpm), while it remains relatively stable within a narrow range at a higher rotational speed (1200 rpm to 1600 rpm). For instance, the peak temperatures recorded at the position c under 800 rpm, 1200 rpm and 1600 rpm are 346.7 °C, 360.7 °C and 360.7 °C under a low welding speed of 90 mm/min. Under a high welding speed of 600 mm/min, the corresponding peak temperatures are 246.8 °C, 263.8 °C and 268.8 °C. This phenomenon can be ascribed to the impact of welding parameters on the axial force of the stirring tool. Pulgarín et al. [35] revealed that the axial pressure of the stirring tool decreases with the increase in rotational speed. Therefore, increasing rotational speed does not necessarily lead to an increase in heat generation. Moreover, a multiple linear regression model is employed to conduct a sensitivity analysis of the influence of various welding parameters on the peak temperature in this study. The formula is described as:

(18) $$T_{peak}=\beta_{0}+\beta_{1}\cdot X_{1}+\beta_{2}\cdot X_{2}+\beta_{3}\cdot X_{3}+\varepsilon$$

where $T_{peak}$ is the peak temperature of the characteristic point, and $\beta_{0}$ is the intercept (constant term), representing the predicted output value when all input parameters are zero. $\beta_{1}$, $\beta_{2}$ and $\beta_{3}$ are the regression coefficients (sensitivity coefficients), indicating the influence of each input parameter on the output. $X_{1}$, $X_{2}$ and $X_{3}$ are the welding speed, rotation speed and the location value (if the point is located in AS, its value is set to 0; otherwise, it is set to 1). ε is the error term. Figure 8b shows the results of the sensitivity analysis. $\beta_{0}$, $\beta_{1}$, $\beta_{2}$ and $\beta_{3}$ are 335.9 °C, −0.18, 0.02 and −11.3, respectively. The coefficient of determination is 0.974, indicating that the welding parameters can explain a significant portion of the peak temperature variation. The calculated results show that the location has the greatest effect, while welding speed and rotation speed have comparatively less influence on the peak temperature of the characteristic points.

It has been reported that the grain sizes on AS and RS are influenced by a combined effect of deformation and temperature. For instance, the grain sizes on AS are typically finer than those on RS due to the more intense dynamic recrystallization induced by both plastic deformation and temperatures [40]. Therefore, the temperature variations between AS and RS are examined in this study. Figure 8c further illustrates the influence of welding parameters on the peak temperature difference at both AS and RS. The temperature difference slightly increases with the rotational speed changing from 800 to 1200 rpm and then decreases at 1600 rpm. For instance, under the welding speed of 90 mm/min, the temperature differences are 12.5 °C, 13.7 °C and 13.2 °C at rotational speeds of 800 rpm (the value was 17.3 °C in the experiment), 1200 rpm and 1600 rpm.

### 4.4. Model Limitations

In this study, several assumptions were made to simplify the modelling process. Specifically, the material was assumed to be isotropic and homogeneous, which may not accurately reflect its actual properties. Additionally, the boundary conditions were idealized, disregarding potential environmental influences such as ambient temperature fluctuations. These limitations could affect the model’s accuracy and applicability in real-world scenarios. Future work could address these issues by incorporating material anisotropy, more realistic boundary conditions and nonlinear effects to enhance the model’s predictive capabilities. In addition, the model used here cannot directly simulate the microstructure variations or mechanical properties due to different thermal cycles during welding. However, the temperature obtained from the moving heat source model in this work can serve as input for other models, such as cellular automata (CA). With the simulated temperature as input, the CA can simulate microstructural evolution based on phase transformation equations, recrystallization equations and other governing equations.

## 5. Conclusions

An integrated approach employing both simulation and experimental methods was performed to understand the temperature field in FSW AZ31 Mg alloys. The influence of welding parameters on the peak temperature was further investigated. The following conclusions are reached:

(1) A modified moving heat source model is proposed by considering the variations in heat generation caused by friction at both the side and bottom surfaces of the tool. The application of this model is further extended to the entire welding process, including the plunging stage. The proposed model accurately simulates the main characteristic of the temperature fields in FSW AZ31 Mg alloys. The largest temperature difference between the simulation value and experimentally measured value is less than 10% at any characteristic point examined.
(2) The temperature distributions at various process of the welding, especially the plunging stage, were revealed by the modified moving heat source model. It was found that the high temperature region moves downward with the tool plunged into the workpiece, and the isothermal surface transforms from an arc shape to a cone shape at the cross-section of the workpiece. Simultaneously, the peak temperature gradually rises to 394.2 °C. The shapes of the isothermal surfaces remain similar during the dwelling-I, stable welding and dwelling-II stages, with the peak temperature reaching 411.4 °C, 484.5 °C and 492.2 °C, respectively. Furthermore, the temperature variations on both AS and RS are asymmetrical with respect to the weld centre.
(3) The influence of welding parameters on the peak temperature of FSW Mg alloys was revealed. In the range of a relatively lower welding speed and rotational speed, the welding temperature rises significantly with both increases in the rotational speed and decreases in the welding speed. By contrast, this effect gradually diminishes within a high range of welding speed and rotational speed.

## Figures and Tables

**Table 1:** Detailed welding parameters.

Welding Speed/(mm/min)	Rotation Speed/rpm
90, 150, 200, 250, 300, 350, 400, 450, 500, 550, 600	800, 1200, 1600

## Data Availability

The raw data supporting the conclusions of this article will be made available by the authors on request.
